# Reaching consensus on the physiotherapeutic management of patients following upper abdominal surgery: a pragmatic approach to interpret equivocal evidence

**DOI:** 10.1186/1472-6947-12-5

**Published:** 2012-02-06

**Authors:** Susan D Hanekom, Dina Brooks, Linda Denehy, Monika Fagevik-Olsén, Timothy C Hardcastle, Shamila Manie, Quinette Louw

**Affiliations:** 1 Department of Interdisciplinary Health Sciences, Division of Physiotherapy, Faculty of Health Sciences, Stellenbosch University, Francie van Zyl Drive, Tygerberg 7505 South Africa; 2 Department of Physical Therapy 160-500 University Avenue, Toronto, Ontario M5G 1V7 Canada; 3 Department of Physiotherapy, The University of Melbourne, Parkville Melbourne, 3010 Australia; 4 Department of Physical Therapy, Sahlgrenska University Hospital, Gothenburg, 413 45, Sweden; 5 Trauma Surgery and Trauma ICU, Inkosi Albert Luthuli central Hospital & University of KwaZulu-Natal 800 Bellair Rd Mayville Durban 4058 South Africa; 6 Department of Health and Rehabilitation Sciences, Division of Physiotherapy, University of Cape Town, Old Main Building, Groote Schuur Hospital, Observatory Cape Town 7925 South Africa

## Abstract

**Background:**

Postoperative pulmonary complications remain the most significant cause of morbidity following open upper abdominal surgery despite advances in perioperative care. However, due to the poor quality primary research uncertainty surrounding the value of prophylactic physiotherapy intervention in the management of patients following abdominal surgery persists. The Delphi process has been proposed as a pragmatic methodology to guide clinical practice when evidence is equivocal.

**Methods:**

The objective was to develop a clinical management algorithm for the post operative management of abdominal surgery patients. Eleven draft algorithm statements extracted from the extant literature by the primary research team were verified and rated by scientist clinicians (n = 5) in an electronic three round Delphi process. Algorithm statements which reached a *priori *defined consensus-semi-interquartile range (SIQR) < 0.5-were collated into the algorithm.

**Results:**

The five panelists allocated to the abdominal surgery Delphi panel were from Australia, Canada, Sweden, and South Africa. The 11 draft algorithm statements were edited and 5 additional statements were formulated. The panel reached consensus on the rating of all statements. Four statements were rated essential.

**Conclusion:**

An expert Delphi panel interpreted the equivocal evidence for the physiotherapeutic management of patients following upper abdominal surgery. Through a process of consensus a clinical management algorithm was formulated. This algorithm can now be used by clinicians to guide clinical practice in this population.

## Background

Postoperative pulmonary complications (PPC) remain the most significant cause of morbidity following open upper abdominal surgery (UAS)[1], despite advances in peri-operative care [2]. PPC's have been defined as "an identifiable disease or dysfunction that is clinically relevant and adversely affects the clinical course"[3]. This umbrella-term includes pneumonia, atelectasis, respiratory failure, bronchospasm and acute exacerbation of COPD [1]. Studies evaluating PPC as outcome do not always specify the specific disease and the criteria used for diagnosis are not consistent [1].

Pre- and postoperative physiotherapeutic treatment forms part of the overall care of major surgical patients to reduce the incidence of PPC [4]. In order to formulate best practice recommendations for the physiotherapeutic management of this population we developed a search strategy to identify empiric evidence published in the last 10 years. Through a systematic search of six databases we identified seven primary research reports [5-11] and six systematic reviews [12-18]. All the primary research reports were included in the reviews. These reports focused on the role of specific physiotherapy techniques used in the postoperative period. This includes the comparative effectiveness of different treatment modalities including ambulation, incentive spirometry, continuous positive airway pressure (CPAP), positive expiratory pressure (PEP) and conventional physiotherapy including deep breathing exercises [5-11]. The methodological quality of the systematic reviews was acceptable scoring at least 8/10 on The Assessment of Multiple Systematic Reviews Tool (AMSTAR). However the reviews were inconclusive. All reviewers commented on the poor quality of the primary research. This included ill defined outcomes [12,15]; heterogeneity of populations and interventions [15]; and the majority of papers not being powered to produce a valid result [12]. Despite the published reports, uncertainties remain. These uncertainties are twofold: Firstly, is the routine application of physiotherapy intervention to all patients following UAS more effective than no intervention in preventing PPC's? Secondly, which physiotherapeutic management options are the most effective in preventing PPC's?

This uncertainty leaves clinicians working in this clinical area partly dependant on their own clinical experience when making decisions regarding individual patient management [19], thus resulting in variation in clinical practice. Variation in clinical practice in turn affects patient outcome and has therefore been one of the driving forces behind the development of evidence based practice. Clinical decisions about patient management incorporate a range of factors, although a necessary element should be the best evidence available, albeit limited. Practical approaches are thus required to assist clinicians in making the optimal management decisions. In recent years, Delphi expert panels have frequently been used in a range of medical fields to assist in the development of evidence based recommendations when only limited or equivocal evidence is available [20-22].

The prophylactic use of physiotherapy to prevent pulmonary complications following abdominal surgery was instituted at the beginning of the 20^th ^century and as such is regarded as a standard of care [4,12]. To justify the routine use of prophylactic physiotherapy after abdominal surgery, we need to be confident of efficacy and the minimal likelihood of harm. The flip side also holds true. Terminating this practice needs to be based on credible and generalisable reports of lack of benefit or increased likelihood of harm. Due to the poor quality of primary research reports systematic reviews have been unable to synthesize evidence, resulting in uncertainty. In order to bridge the gap between evidence and clinical practice, the aim of this paper is to develop an evidence based clinical management algorithm for the management of patients following abdominal surgery through a Delphi process of consensus.

This work represents a section of a larger study involving the role of physiotherapy in Intensive Care Units (ICU). A comprehensive evidence-based physiotherapeutic framework for the management of adult patients, admitted to a surgical ICU, was developed. A Delphi panel of twenty-seven identified research clinicians in the area was convened to validate the framework. Experts were divided into sub-groups on the basis of their publication record and were only required to comment on specific algorithms. This paper reports on the process followed by the abdominal surgery sub-group.

## Methods

**Figure 1:**
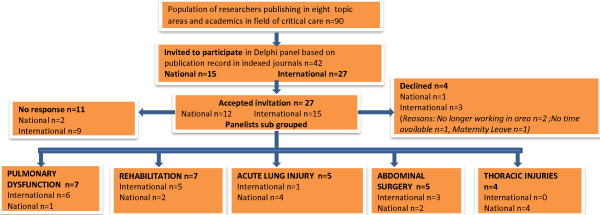
**Delphi panel allocation**.

***Ethical approval ***was provided by the ethics committee of Stellenbosch University and all participants provided informed consent. ***Study Structure: ***A three round Delphi process was used to develop the clinical management algorithm. ***Selection of Delphi panelists***: Authors of peer-reviewed publications relating to the prevention of pulmonary complications following abdominal surgery, indexed in *Medline, CINAHL, Web of Science, PEDro, Science Direct, Cochrane or TRIP *or published in the SAJP or SAJCC were eligible to participate. Researchers were excluded if they were not electronically contactable, or declined the invitation (Figure 1).

### Instrumentation

An interactive website (http://www0.sun.ac.za/Physiotherapy_ICU_algorithm) linked to a password- protected database was developed to distribute information and collate responses from the Delphi panel. The website contained the original papers, the evidence synthesis reports (data extraction and quality assessment reports of systematic reviews), draft best practice recommendations and draft algorithm statements. The process described by Lobach and Kerner [23] was used to convert recommendations into algorithm statements. SH and QL developed the draft algorithm statements through deliberation and discussion of the information obtained during the data extraction process. Eleven draft algorithm statements were formulated. These statements were grouped into four categories. **Category A **included statements related to the coughing procedure; **Category B**, criteria for mobilization; **Category C**, breathing techniques; and **Category D**, mobilization options (Table 1).

**Table 1:** Verification and rating of algorithm statements

	REVISED STEPS AFTER ROUND 1	n	RATING ROUND 2 MEDIAN (SIQR)	RATING ROUND 3 MEDIAN (SIQR)
**COUGHING AND PROCEDURE ADOPTED TO FACILITATE COUGHING**				
Teach pt to huff/cough with wound support	1. Teach patient to huff/cough with wound support. Use following strategies to facilitate procedure: deep breathing, PEP, high-pressure PEP and CPAP in combination with FET (or adjusted autogenic drainage).	5	2.0 (0.5)	1.0 (0.0)
If unsuccessful: suction patient through mouth piece	2. If all else fails include suctioning as a possible management strategy for removing secretions	5	2.0 (0.5)	2.0 (0.0)
	3. Use nebulization as a management option for the removal of secretions	5	3.0 (0.5)	3.0 (0.0)
Position pt in high sitting over the side of the bed	4. Position the patient in a stable, supported upright sitting position with a goal of positioning the patient out of bed to facilitate removal of secretions	5	1.0 (0.0)	1.0 (0.0)
**CRITERIA FOR MOBILIZATION**				
At rest pt is presenting stable blood pressure and heart rate with less than 8/10 rating on pain scale	5. Perform a clinical evaluation of pain level	5	3.0 (0.0)	3.0 (0.0)
At rest pt is presenting with no dyspnoea	6. At rest dyspnoea does not exceed 1 on MBS.	5	3.0 (0.5)	3.0 (0.0)
	7. Ensure sufficient pulmonary reserve (Oxygenation level PaO_2_:FiO_2 _> 40 kPa/300 mmHg) before initiating mobilization.	5	2.0 (1.5)	2.0 (0.0)
	8. Motor block assessment in patients receiving epidural analgesia	5	2.0 (0.5)	2.0 (0.0)
**BREATHING TECHNIQUES**				
Position pt in high sitting over the side of the bed/Long sitting in bed	Incorporated into steps 4 and 16			
	9. Prescribe frequent breathing exercises-the goal is at least five maximum breaths every waking hour.	4	1.0 (0.25)	1.0 (0.0)
Use any of the following techniques based on pt performance: PEP mask; IPPB; PEEP Bottle; IS	10. Present breathing technique choice in the following hierarchy: DBE's followed by PEP mask or bottle; then IS and IPPB as the least likely choice.	4	2.0 (1.0)	2.0 (0.13)
	11. Deep breathing exercises (pursed lips breathing; inspiratory hold) are the first choice of breathing exercises with PaO_2_:FiO_2 _> 300 mmHg.	4	2.0 (0.88)	2.0 (0.0)
	12. In the presence of persistent post operative hypoxaemia (PaO_2_:FiO_2 _< 300 mmHg) initiate CPAP.	4	2.0 (0.63)	2.0 (0.0)
**MOBILIZATION**				
Pt must reach at least one of these goals with each treatment session: Sit out of bed; Walk 5 m; 15 m; 30 m with assistance; Walk 30 m without assistance.	13. Perform activities at dyspnoea intensity of 6 on the MBS.	5	1.0 (1.0)	1.0 (0.0)
Progression based on walking intensity of 6/10 on Borg Scale	Incorporated into step 13			
Active dorsiflexion while in bed at least 20 times every waking hour	14. Active dorsiflexion while in bed at least 20 times every waking hour	5	4.0 (0.5)	4.0 (0.0)
Frequency: Days one and two (three times/day)	15. An intensive mobilization protocol that includes walking and stair climbing should be performed at least once daily with the goal of three times per day.	5	1.0 (0.5)	1.0 (0.0)
	16. Have patient sitting out of bed for a minimum of one hour twice daily AND walking at least 5 m as the goal on the first post operative day	5	1.0 (0.5)	1.0(0.0)

CPAP: Continuous positive airway pressure; DBE: Deep breathing exercises; FET: Forced expiratory technique; FiO2: Fraction of inspired oxygen; IPPB: Intermittent positive pressure breathing; IS: Incentive spirometry; MBS: Modified Borg Scale; PEP: Positive expiratory pressure; pt: patient.

**Figure 2:**
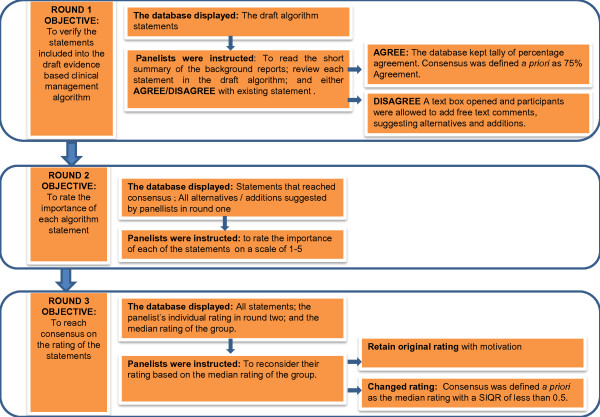
**Verification and rating of the algorithm statements**. SIQR Semi inter quartile range.

The functionality of the database changed in relation to the specific round of the three round Delphi process (Figure 2).

### Delphi study procedure

Each Delphi round lasted two weeks. During this time panelists had unlimited access to the database and an opportunity to add anonymous free text comments. Following each round, a summary of responses not registered on the database was communicated electronically to individual panelists by the chief investigator (SH) to provide an opportunity to complete responses. This individual communication was concerned with logistical issues and not related to content.

### Data Analysis

The median (semi-interquartile range, SIQR) was calculated for each algorithm statement. Consensus for algorithm statements was defined *a priori *as a SIQR < 0.5.

### Formulation of the final algorithm (Figure 3)

All statements which reached consensus were collated into a clinical algorithm using descriptors based on the median rating (http://www0.sun.ac.za/Physiotherapy_ICU_algorithm).

**Figure 3:**
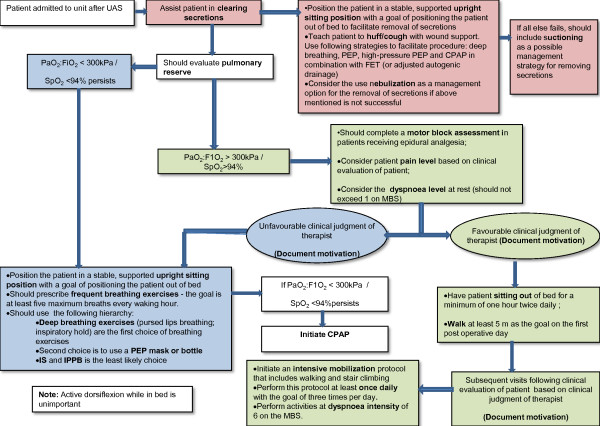
**A consensus clinical algorithm for the management of patients following upper abdominal surgery**. CPAP: Continuous positive airway pressure; DBE: Deep breathing exercises; FET: Forced expiratory technique; FiO2: Fraction of inspired oxygen; IPPB: Intermittent positive pressure breathing; IS: Incentive spirometry; MBS: Modified Borg Scale; PEP: Positive expiratory pressure.

## Results

The five panelists allocated to the abdominal surgery panel were from Australia, Canada, Sweden and South Africa and included four physiotherapists and a trauma surgeon. The draft statements were edited and five additional statements were formulated (Table 1).

In **Category A**: the panel agreed after the second round that it is essential to position the patient in a stable, supported upright sitting position and teach the patient to huff/cough with wound support as soon as possible following surgery. The panel agreed on the rating of all the statements in this category (4/4) after the third round.

The importance of coughing is supported by strong evidence from the systematic reviews [12,16]. However the panel went further and decided it would be essential also to include management strategies to ensure effective secretion removal. These strategies are based on the collective clinical experience of this group. It includes positioning the patient out of bed and using interventions such as deep breathing, positive expiratory pressure (PEP), high-pressure PEP and CPAP in combination with forced expiration technique (or adjusted autogenic drainage). If these approaches failed, the inclusion of suctioning was rated as very important.

In **Category B**: two additional statements were formulated, and the structure of two statements was edited based on feedback from the panel. The structural editions of statements were related to being less prescriptive for example *"Initiate mobilization when patient is presenting stable blood pressure and heart rate with less than 8/10 rating on pain scale at rest" *was changed to *"Perform a clinical evaluation of pain level"*. The panel agreed that prophylactic physiotherapy intervention following abdominal surgery was essential, but that the choice of intervention could either be mobilization or breathing exercises, but does not need to include both options.

The Delphi panel agreed that early directed mobilization was the first management option to consider. This choice is supported by the literature [16] and in line with the accepted physiological benefit of being up and mobile [24,25]. The panel chose to rate both the intensity and frequency of a mobilization protocol as essential to the effectiveness of a management algorithm. This is an interesting result, since few studies have been undertaken that examine the effectiveness of mobilization as a therapeutic option for reducing PPC's. Although one study reported that time spent upright in patients after abdominal surgery was low at an average total of three minutes on the first postoperative day [26]. It is possible that this result and results from other patient populations may be influencing this panel consensus. There is a growing body of evidence that early mobility influences outcome in general ICU [27-29] and randomized trials supporting the role of mobilization after cardiac surgery [30]. Further research is needed to support the views of the panel in relation to mobilization practices in an abdominal surgical population.

In **Category C: **three additional statements were formulated and the content and structure of the remaining statement was edited. The statements that were added were related to the frequency of breathing exercises and the indication of which breathing techniques to include. The structural change to the one statement was to direct breathing exercise preference. The panel reached consensus on the rating of all statements after the third round. Prescribing frequent breathing exercises was the only statement rated essential in this category. While the evidence suggests that there is no difference in the effectiveness of the type of breathing exercises used to prevent PPC's in this population [12,16], the panel agreed to include a hierarchy of breathing methods into the algorithm. The GRADE system [31] whereby the potential benefit (outcome) is weighed against the burden (financial and time related) of application and potential harm was used as a basis for the hierarchy. This hierarchy can be used as a guide by clinicians when choosing a breathing exercise. Deep breathing exercises (DBE)-using pursed lips breathing or inspiratory hold-was the first choice expressed through this Delphi process [32,33]. This technique is not therapist or device dependent and was therefore accepted as the first choice.

The second choice agreed by the panel was positive expiratory pressure (PEP) by mask or bottle. This method is routinely used in the Nordic countries, and has been evaluated in several settings [10,34]. While the PEP mask is costly and not always available, the same effect can be achieved with a blow-bottle technique and is thus regarded as a cheaper alternative of the PEP mask. The least likely choice is incentive spirometry (IS) [18] followed by intermittent positive pressure breathing (IPPB). Both these techniques are dependent on specialized equipment and therefore costly to the patient. Two systematic reviews reported no added benefit to deep breathing exercises [13,14,35]. In addition, IPPB would be the last choice because abdominal distention has been reported as potentially harmful and the technique is therapist dependent [14,18].

Thirdly, the panel agreed that it is essential that the breathing exercises which are prescribed should be performed frequently. This decision is based on the clinical experience of this expert panel as no studies were identified through this process which could inform on the frequency of breathing exercises in this population. Studies investigating other populations, as well as the short-lived physiological effect of breathing exercises, could have influenced this panel's judgment [36].

Finally, based on the literature, this Delphi panel was in accord that continuous positive airway pressure (CPAP) was useful as an adjunct to deep breathing exercises and as a preventative strategy in reducing complications [37]. In the presence of persistent hypoxaemia which is unresponsive to first line physiotherapy management, there is moderate quality evidence to suggest that CPAP intervention will reduce the risk of PPC's [37].

In **Category D**, two statements were reformulated into a single statement; while one statement was reformulated into two separate statements. The importance of the clinical judgment of the therapist in initiating mobilization is highlighted by the fact that the panel did not rate the inclusion of specific criteria as essential to the success of the algorithm. The two criteria that were rated as very important-the assessment of motor block in patients receiving epidural analgesia and the evaluation of pulmonary reserve-could guide this clinical decision. The panel rated the inclusion of active dorsiflexion while in bed as unimportant.

## Discussion

Through a Delphi process of consensus this international panel formulated four statements which could form the interim basis for clinical practice in this population. Firstly, it is essential to position patients in a stable, supported upright sitting position and to teach them to huff/cough with wound support as soon as possible post operatively. Secondly the panel agreed that prophylactic physiotherapy intervention following abdominal surgery was essential. The choice of intervention could either be mobilization or breathing exercises, but does not need to include both options. Thirdly, it is essential that when breathing exercises are prescribed they should be performed frequently. Finally, this Delphi panel was in accord that CPAP was a useful adjunctive strategy to reduce complications in selected patients [37].

The importance of clinical expertise in the clinical decision-making process has been reported [38,39]. However, ways in which this expertise can be defined and incorporated into evidence-based practice still need to be explored, specifically in grey areas of clinical practice. This report demonstrates that convening a Delphi expert panel could be a pragmatic way in which to provide directive to clinicians when empiric evidence is lacking or equivocal. Similar uses of this methodology have been reported in a range of medical fields to assist in the development of evidence-based recommendations, in the presence of limited or equivocal evidence [40,41]. We used a novel approach in the formulation of the clinical management algorithm statements. Due to the gaps in the evidence base we incorporated a rating system for the algorithm statements. We did not discard statements which were not rated essential by the panel. We argued that lack of evidence does not equate to lack of benefit and therefore the management options should still be available for use. This facilitated the development of a hierarchical framework of available options for clinicians to consider when making a decision on the management of individual patients. In addition this report also highlights the use of a Delphi methodology as a robust way of reaching consensus on the formulation of best practice recommendations. The anonymity of the panellists throughout the process allowed all views to be considered, and provided panellists the opportunity to change their opinions based on the merit of the arguments presented. This process is in line with efforts by the World Health Organization to improve the science of guideline development [42].

We recognize that decisions made regarding the compilation of this Delphi panel could limit the external validity of the algorithm [43]. However the decision to limit the panel to researchers in this field was deliberate because it was expected that these researcher clinicians would be well informed on the clinical decision making factors pertaining to the management of patients following abdominal surgery [44]. We recognize that this decision necessarily implies the potential of a vested discipline specific interest in the prophylactic use of physiotherapy intervention. The inclusion of the trauma surgeon and the international profile of the panelists should alleviate some concerns. Secondly, the majority of reports published in this field over the past 10 years have focused on secondary synthesis of primary studies. This could explain the small number of researchers who qualified for participation. Finally, the sample was limited to researchers with a track record in the specific subject area. New researchers in this specific area of interest were therefore not included. These decisions are in line with current recommendations for Delphi panel composition [43,44].

The interpretation of current available evidence combined with the clinical expertise of this international panel presented in this paper can now also form the basis for primary research in this population. The potential for type II errors in available research data for physiotherapy interventions following upper abdominal surgery is highlighted in this report. A beta error results in an erroneous conclusion that there are no differences between two treatment groups when it does exist [45]. This erroneous conclusion of underpowered studies has been reported across various disciplines [46-49]. Methodologists [50] argue that it is more likely within medical science that small but clinically meaningful difference exists between treatment groups. To detect these differences would then imply that large trials are required. Some methodologists have argued that the results of underpowered studies can be pooled in a meta-analysis and that all trial information is therefore of value [50,51]. However, it has not been possible to use meta-analysis tools to combine the results of the many underpowered studies in this field, due to heterogeneity in populations, interventions investigated and outcomes measured [12].

Going forward, there is an urgent need for sufficiently powered clinical trials which report on the effectiveness of postoperative physiotherapy as a management option [52,53]. To do this, rigorous clini-metric development of outcomes such as post operative pulmonary complications and length of postoperative hospital stay needs to be undertaken.

## Conclusion

Due to the poor quality of the primary research, and the danger of beta errors in this body of work, uncertainty about the value of routine physiotherapy in the prevention of pulmonary complications following abdominal surgery, remain. Through a process of consensus, the international Delphi panel interpreted the equivocal evidence and, combined with the collective expert opinion, formulated an algorithm. This algorithm now provides clinicians with a hierarchical framework within which optimal clinical management decisions can be made at the bedside.

## Competing interests

The authors declare that they have no competing interests.

## Authors' contributions

SH and QL generated the draft algorithm statements based on a systematic review of the literature. SH managed the three stages of the Delphi process. DB, LD, MF-O, TH, SM participated in formulating and rating evidence based statements. SH and QL participated in the design of the study and performed the statistical analysis. SH and QL conceived of the study, and participated in its design and coordination. All authors contributed to the draft manuscript. All authors read and approved the final manuscript.

## Pre-publication history

The pre-publication history for this paper can be accessed here:

http://www.biomedcentral.com/1472-6947/12/5/prepub
